# Measuring the effects of mind wandering in people with insomnia: A driving simulator study

**DOI:** 10.3389/fnins.2022.944096

**Published:** 2022-08-17

**Authors:** Lin Xu, Yingying Yan, Hongming Dong, Dandan Qiao, Yanyan Liu, Junfang Tian, Zhu Ai, Rong Xue

**Affiliations:** ^1^Department of Neurology, Tianjin Medical University General Hospital, Tianjin, China; ^2^College of Management and Economics, Tianjin University, Tianjin, China; ^3^Department of Geriatrics, Beijing Luhe Hospital, Capital Medical University, Beijing, China; ^4^Department of Neurology, Liuzhou Worker’s Hospital, Liuzhou, China

**Keywords:** people with insomnia, mind wandering, cognitive distraction, driving simulator, driving performance

## Abstract

**Purpose:**

Studies have shown that individuals with insomnia experience more frequent and longer episodes of mind wandering (MW) while driving. However, the effect of the interaction between insomnia and MW on driving behavior is not fully understood. This study aimed to gain deeper insights into the relationships among insomnia, MW, and driving behavior.

**Patients and methods:**

Forty-two participants (21 diagnosed with insomnia and 21 controls) were recruited, and subjective sleep quality and cognitive function were assessed. A driving simulator experiment with a within-subject design was performed, involving two distraction tasks (no-distraction task versus MW task) and two driving scenarios (lane-keeping versus lane-changing).

**Results:**

In the lane-keeping scenario, there was no significant between-group difference (people with insomnia and controls) in longitudinal driving performance for the no-distraction task, although the interaction between MW and insomnia significantly increased drivers’ longitudinal control variation. Correlation analysis confirmed that longitudinal driving performance was positively correlated with sleep quality and the cognitive level. Unlike longitudinal driving performance, lateral driving performance was significantly weaker in people with insomnia than in controls under both distraction tasks. In the lane-changing scenario, although there was no between-group difference in driving performance, the MW task led to significant changes in driving performance within each group compared with the no-distraction task, and these findings were associated with cognitive function, but not with sleep quality.

**Conclusion:**

These findings show that insomnia and MW combined can lead to reduced driving performance. Further research is needed to elucidate the factors that influence this phenomenon.

## Introduction

Sleep disorders are a growing challenge to road traffic (Perrier et al., 2014). A previous study reported that 16.9% of drivers had at least one sleep disorder, while 8.9% experienced an episode of sleepiness at least once a month while driving and 0.42% reported at least one sleepiness-related driving accident (Philip et al., 2010). On average, sleepiness accounts for 20% of all traffic accidents in developed countries and costs the United States at least $12.5 billion in monetary losses per year (Shekari Soleimanloo et al., 2017). Sleepiness is mostly caused by sleep disorders, such as insomnia, which is a condition that is becoming increasingly prevalent (Smallwood et al., 2011). A study found that people with insomnia drove an average of 8,948 km per year for personal purposes and had a three-fold higher risk of major traffic accidents compared with that posed by normal sleepers (Léger et al., 2006).

However, the issues surrounding driving safety associated with insomnia have not received widespread attention. Previous works have shown that insomnia can cause cognitive dysfunction and is especially likely to impair the cognitive domains of vigilance, attention, and reaction time, which in turn can affect an individual’s daytime functioning and behaviors, including cognitively demanding driving behaviors (Léger et al., 2006; Jeon et al., 2014; Miyachi et al., 2021). Ahlström et al. found that sleep-deprived participants showed low lambda responses, which led to a general decline in cortical responsiveness to incoming visual stimuli; furthermore, low lambda responses were also associated with more line crossings while driving (Ahlström et al., 2020). According to previous works, insomnia caused drivers to exhibit a higher standard deviation of lateral position (SDLP) and standard deviation of speed (SDS), as well as to exhibit a greater difficulty in predicting crash risk (Bhattacharya et al., 1987; Perrier et al., 2014).

In addition to insomnia, frequent mind wandering (MW) while driving has also attracted growing attention from the academic community. According to one study, 85.2% of drivers reported experiencing MW while driving and that MW accounted for an average of 34.74% of their trips (Berthié et al., 2015). In academic research, MW generally is referred to the internal cognitive distraction caused by a shift of the driver’s attention from primary driving tasks to internal mental information in the absence of external sources of auditory or visual interference (Smallwood and Schooler, 2006; Martens and Brouwer, 2013). Current research on MW while driving is still relatively scarce, and there are limited information concerning the effects of MW on driving performance. A car-following experiment with normal participants revealed that MW had virtually no negative impact on the drivers’ vehicle control compared with those driving attentively, and drivers tended to focus their visual attention on the road ahead (He et al., 2011). However, some studies have stated that MW while driving decouples attention from visual and auditory perceptions, which compromises the drivers’ ability to absorb information from the environment, thereby negatively interfering with driving and threatening driving safety (Burdett et al., 2016; Nijboer et al., 2016). An interview study found that 17% of traffic crashes, in which the driver was thought to be responsible, were associated with intense MW while driving (Galéra et al., 2012). In addition, drivers’ MW led to faster driving speeds, reduced lateral control, shorter distances between vehicles, and a longer reaction time in response to sudden events (Lemercier et al., 2014; Albert et al., 2018).

Low sleep quality can also increase the frequency of MW in drivers (Smallwood et al., 2011; Walker and Trick, 2018). A previous research found that poor sleep quality was associated with MW (Carciofo et al., 2014; Stawarczyk and D’Argembeau, 2016). Especially, individuals with sleep disorders were 29% more likely to be inattentive while driving compared with normal sleepers (Bharadwaj et al., 2021). However, only a few studies have focused on cognitively distracted driving behaviors in people with insomnia during MW. In fact, driver inattention and sleep deprivation contribute to approximately 5–25% of traffic accidents, as well as approximately 20% of fatal and serious crashes (Ferreira et al., 2019). Previous works have suggested that an individual’s ability to maintain attention and inhibit distraction is primarily controlled by the prefrontal cortex, although this region has been shown to be susceptible to the negative effects of sleep deprivation (Maquet, 2000). A selective attention task observed that people with insomnia were more prone to distraction and that insomnia symptom severity was positively correlated with participants’ distractibility (Miller et al., 2021). Moreover, a study analyzed real-world driving data and found that for every 10% increase in driving time, the frequency of sleepiness and distraction alerts increased by 15 and 10%, respectively (Anderson and Horne, 2006). Especially, sleepiness and the number of distraction alerts increased as the duration of continuous driving increased. Moreover, a driving simulated experiment found that sleepy individuals were more likely to be distracted, especially when performing a monotonous task, and that sleep deprivation led to a four-fold increase in the number of prolonged distractions (Carpenter and Andrykowski, 1998), that is, distractions caused by sleep deprivation lasted longer. In summary, distractions occur more frequently and persist for longer with insomnia-induced sleepiness, while sustained attention is an ability required for driving and has a significant impact on driving safety.

At present, several studies have separately analyzed the effect of insomnia or MW alone on driving performance, although only a few have focused on the interaction effect of the two on driving performance. To fill this gap in knowledge, this study aimed to investigate the driving behavior of people with insomnia during cognitive distraction caused by MW. To conduct the study, 42 participants were recruited for a driving simulator experiment, including patients diagnosed with insomnia and normal sleepers as controls. The experiment adopted a within-group design, involving two distraction tasks (no-distraction task versus MW task) and two driving scenarios (lane-changing versus lane-keeping).

## Methods

### Experimental design

#### Subjective sleep quality assessment

Before the driving simulator experiment, participants were asked to complete the Pittsburgh Sleep Quality Index (PSQI) questionnaire, which served as an indicator of subjective sleep quality. The PSQI is suitable for evaluating an individual’s sleep status in the last month, with a total score ≤8 points indicating normal sleep and >8 points indicating the presence of sleep disorders (Nasreddine et al., 2005).

#### Cognitive function assessment

Participants’ cognitive function was evaluated using the Montreal Cognitive Assessment (MoCA) score, which has been widely used to assess cognitive functions, including attention and concentration, executive function, memory, language, visuospatial skills, abstraction, and orientation (Brookhuis et al., 1990).

#### Driving scenarios

The vehicles were driven on a straight road section comprising three lanes with a speed limit of 40 km/h. Participants were instructed to adhere to the speed limit as accurately as possible. Two different driving scenarios were investigated (Lee et al., 2003; Rodrick et al., 2013): (1) a lane-keeping scenario, in which the vehicle was always driven in the middle lane; and (2) a lane-changing scenario, in which the vehicle was initially positioned in the middle lane, with turn signs presented every 150 m, and participants were instructed to change lanes according to the turn signs.

#### Distraction tasks

The distraction tasks comprised a no-distraction task and an MW task. The MW task involved an intelligence quotient (IQ) test (Martens and Brouwer, 2013). Before MW driving, the researcher asked the participants IQ questions that required memory skills and were difficult to calculate directly. For example, if one bottle of water could be bought for one dollar and two bottle caps could be exchanged for one bottle of water, then, how many bottles of water can you buy with 11 dollars? Participants were instructed to remember and think about these questions while driving, and at the end of this experimental scenario, they were asked to report their calculation process and results. These questions were expected to occupy the driver’s cognitive resources as they required the driver to engage their memory function and to perform calculations while driving, in an attempt to encourage MW while driving.

### Participants

In total, 42 participants were recruited for this experiment after excluding individuals with the following characteristics: (1) the presence of organic heart disease or arrhythmia, thyroid disease, and chronic renal failure, a history of psychiatric disease (e.g., major depression), or a history of neurological disease (e.g., stroke, brain tumor, and traumatic brain injury); (2) a tendency for motion sickness; (3) a history of alcohol or drug abuse; (4) less than 3 years of driving experience or inability to drive; (5) pregnant or breastfeeding; and (6) a history of shift work or jet lag in the past month.

All participants were divided into the control group (CG) and insomnia group (IG) based on the diagnosis of physicians at the Sleep Disorder Specialist Clinic of the Tianjin Medical University General Hospital. All participants in the CG were normal sleepers who did not meet the International Classification of Sleep Disorders (ICSD-3) diagnostic criteria for chronic insomnia disorder, with PSQI scores ≤8 points. All participants in the IG were diagnosed with chronic insomnia and met the ICSD-3 diagnostic criteria for chronic insomnia disorder, with PSQI scores >8 points and a history of taking zolpidem.

Both CG and IG comprised 21 participants. All participants had at least 3 years of driving experience; drove >3,000 km per year on average; had at least 9 years of education; had no difficulty with reading, writing, or communication; and did not consume alcohol, coffee, strong tea, or other beverages that can affect sleep on the day of and the day before the experiment. Participants provided written informed consent and participated voluntarily in this study. Table 1 shows the descriptive statistics of the participants’ information.

**TABLE 1:** Driver characteristics.

	CG	IG
	N	N
Gender		
Male	**14**	**15**
Female	**7**	**6**
	**Mean (SD)**	**Mean**
Age (years)	**40.10 (11.93)**	**41.05 (SD = 10.24)**
Driving experience (years)	**11.52 (SD = 3.10)**	**12.33 (SD = 8.007)**

### Experimental procedure

This study was approved by the Medical Ethics Committee of General Hospital of Tianjin Medical University. Before conducting the formal experiment, participants were asked to fill out questionnaires (including a questionnaire on personal information, the PSQI, and the MoCA). Next, a brief training session on using the driving simulator was provided to the participants with a uniform set of instructions. Participants were asked to practice driving for 10 min to familiarize themselves with the operation of the experimental apparatus and environment. The formal experiment included four combinations of distraction tasks and driving scenarios presented in the following order: a no-distraction task in a lane-keeping scenario, an MW task in a lane-keeping scenario, a no-distraction task in a lane-changing scenario, and an MW task in a lane-changing scenario. Participants were required to drive for approximately 10 min for each combination. The experiment was conducted at Tianjin University using a Japanese FORUM8 driving simulator (FORUM8, Tokyo, Japan) with a data acquisition frequency of 20 Hz.

### Data analysis

There were three types of data that were analyzed in this paper: the differences in driving performance between the two groups (CG and IG); within-group differences in driving performance between the two distraction tasks in the same driving scenario; and the correlation between driving behavior and sleep quality or cognitive function.

Several previous studies have used methods, such as analysis of variance, to analyze similar issues, which usually assume that the overall population follows a normal distribution. However, data collected did not fully satisfy the normality and homogeneity requirements, and hence, they were analyzed using non-parametric tests, which make inferences concerning the shape of the overall distribution when the overall variance is unknown or poorly known. Specifically, the Mann–Whitney *U* test was performed to analyze the differences in driving performance between the two groups (CG and IG); the Wilcoxon test was performed to analyze within-group differences in driving performance between the two distraction tasks in the same driving scenario; and Spearman’s correlation analysis was performed to analyze the correlation between driving behavior and sleep quality or cognitive function. The level of significance was set at *P* < 0.05.

## Results

### Sleepiness and cognitive data

Both groups of participants completed the PSQI questionnaire as a measure of subjective sleepiness, and these scores were evaluated using the Mann–Whitney *U* test. The PSQI scores were higher in the IG (mean = 13.43, SD = 2.38) than in the CG (mean = 3.48, SD = 2.87), and the difference was significant (*P* < 0.001).

In terms of cognitive function, the total MoCA scores were higher in the CG (mean = 28.38, SD = 1.09) than in the IG (mean = 27.10, SD = 1.60), and the difference was significant (*P* = 0.002), indicating a decline in the cognitive function of the IG compared with the CG.

### Driving performance

#### Lane-keeping scenario

The driving performance of both groups was measured under a no-distraction task versus an MW task, which included both longitudinal and lateral measures.

**FIGURE 1:**
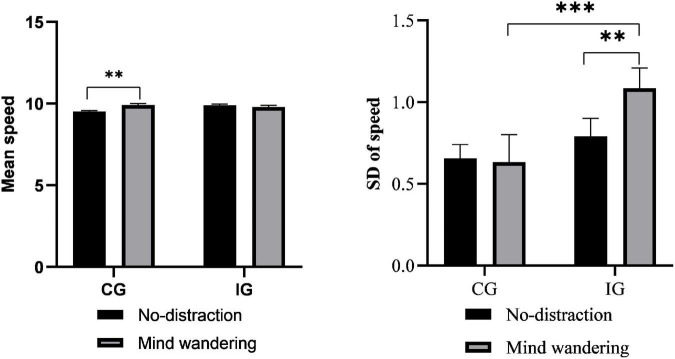
The effect of mind wandering (MW) on speed. Error bars represent the standard error. ***P < 0.001; **P < 0.01; *P < 0.05. MW, mind wandering; SD, standard deviation.

First, there were no significant differences between the two groups in any of the longitudinal control measures under the no-distraction task. In contrast, in the MW task, the Mann–Whitney U test revealed a significant between-group difference in driving performance, with the IG showing a higher SDS than the CG (*P* = 0.001), as shown in Figure 1. In addition, the differences in driving performance between the two distraction tasks were analyzed for each group. The results revealed a significant effect of MW within the IG as indicated by a significantly higher SDS for the MW task compared with the no-distraction task (*P* = 0.019). Despite the absence of between-group differences in the mean speed, there was a significant difference in the mean speed between the two tasks within the CG, which showed a significantly higher mean speed in the MW task compared with the no-distraction task (*P* = 0.009).

**FIGURE 2:**
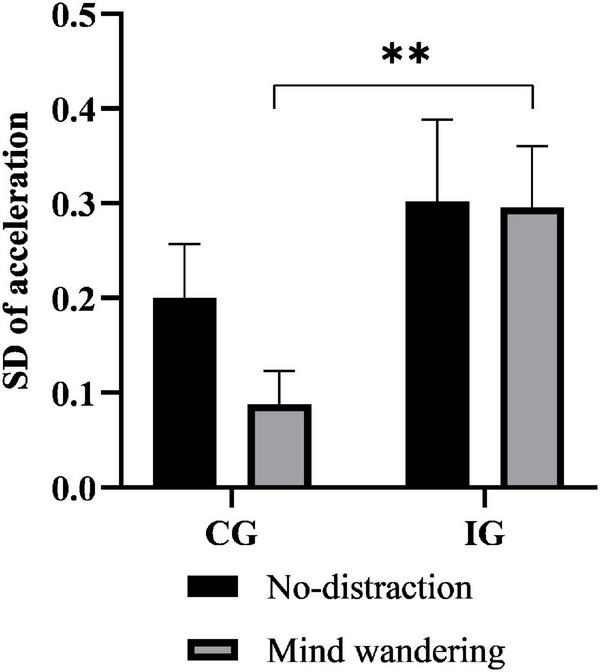
The effect of mind wandering (MW) on acceleration. ***P < 0.001; **P < 0.01; *P < 0.05. MW, mind wandering; SD, standard deviation; IG, insomniac group; CG, control group.

**FIGURE 3:**
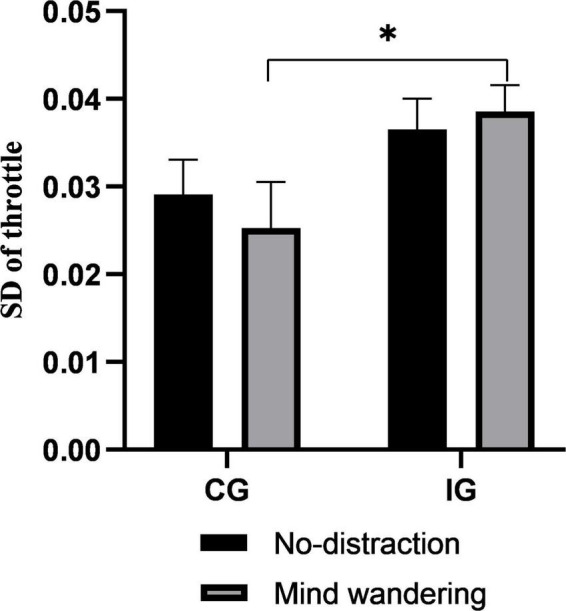
The effect of mind wandering (MW) on the throttle. ***P < 0.001; **P < 0.01; *P < 0.05. MW, mind wandering; SD, standard deviation; IG, insomniac group; CG, control group.

As shown in Figure 2, although both groups exhibited a decrease in the SD of acceleration to some extent under the MW task compared with the no-distraction task, this measure was still significantly higher in the IG than in the CG under the MW task (*P* = 0.007). Similarly, the SD of the throttle value was significantly higher in the IG than in the CG (*P* = 0.013) (Figure 3).

**FIGURE 4:**
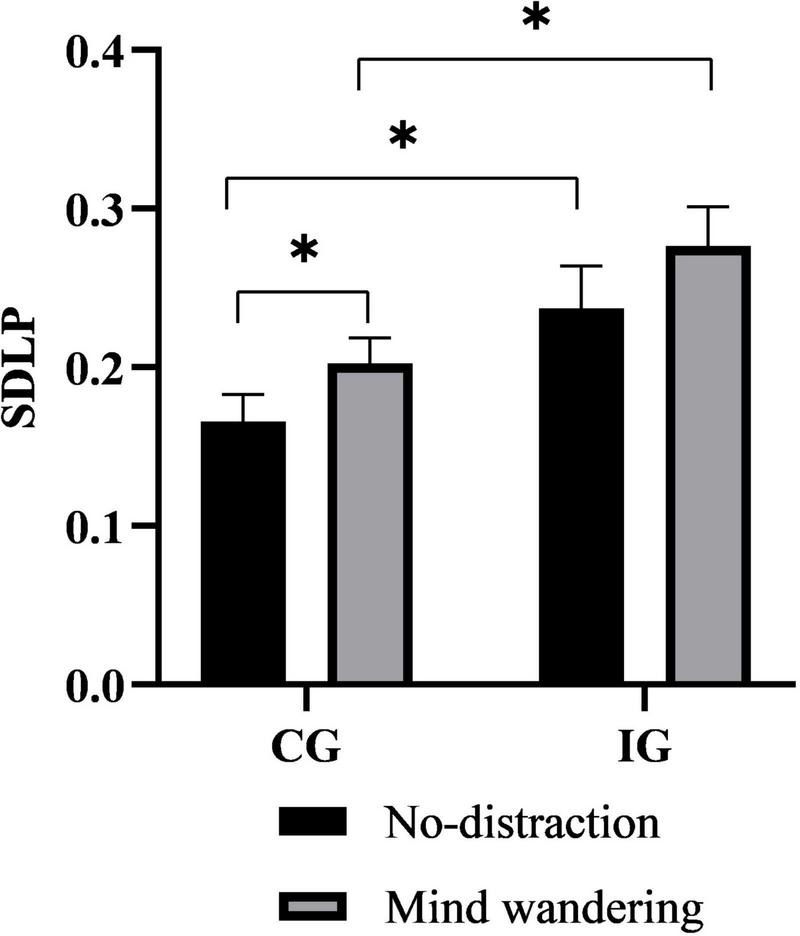
The effect of mind wandering (MW) on the SDLP. ***P < 0.001; **P < 0.01; *P < 0.05. MW, mind wandering; SDLP, standard deviation of lateral position; IG, insomniac group; CG, control group.

Second, the results on lateral control measures indicated that unlike the longitudinal measures, the SDLP was significantly higher in the IG than in the CG, regardless of whether participants were performing the no-distraction task or MW task (*P* = 0.034 and.016, respectively), as shown in Figure 4. Moreover, the Wilcoxon test revealed that the SDLP of the CG was significantly higher in the MW task than in the no-distraction task (*P* = 0.039).

### Lane-changing scenario

**FIGURE 5:**
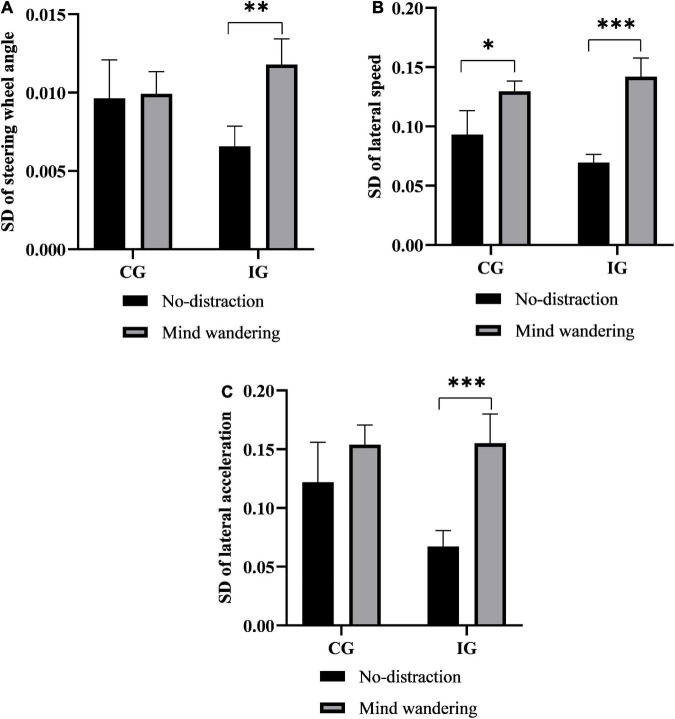
The effect of MW on lane-changing control. **(A)** SD of steering wheel angle. **(B)** SD of lateral speed. **(C)** SD of lateral acceleration. ***P < 0.001; **P < 0.01; and *P < 0.05. MW, mind wandering; SD, standard deviation; IG, insomniac group; CG, control group.

First, there were no significant between-group differences in lane-changing control measures irrespective of the task. Next, the Wilcoxon test was performed to determine whether there were within-group differences in any lane-changing control measures. The results showed that the SD of the steering wheel angle, SD of lateral speed, and SD of lateral acceleration in the IG increased significantly in the MW task compared with the no-distraction task (*P* = 0.006, *P* < 0.001, and *P* < 0.001, respectively). Similarly, the SD of lateral speed in the CG under the MW task was significantly higher than that under the no-distraction task (*P* = 0.023) (Figure 5).

### Relationship between sleepiness and driving performance

Spearman’s correlation analysis was performed between the PSQI scores and driving measures in the MW task to determine whether subjective sleepiness was associated with driving performance. Table 2 lists the significant results of the correlation analysis. Participants’ PSQI scores were positively correlated with the SDS, SD of the throttle, and SD of acceleration in the MW task in the lane-keeping scenario. However, such significant correlations were absent in the lane-changing scenario.

**TABLE 2:** Correlation between the PSQI scores and driving measures.

	PSQI	
	*R*	P
SDS	0.560	0.000
SD of throttle	0.456	0.002
SD of acceleration	0.460	0.001
SDLP	0.468	0.002

SD, standard deviation; SDS, standard deviation of speed; SDLP, standard deviation of lateral position; PSQI, Pittsburgh Sleep Quality Index.

### Relationship between cognition and driving performance

The correlations between the participants’ cognitive level and driving performance in the MW task are presented in Table 3. The MoCA scores were negatively correlated with the SDS, SD of the throttle, SD of acceleration, and SDLP in the MW task in the lane-keeping scenario, indicating that a higher cognitive level was associated with better driving control in the lane-keeping scenario. In the lane-changing scenario, the participants’ MoCA scores were negatively correlated with the SD of lateral acceleration, indicating that under the interference of MW, the participants’ cognitive level was positively correlated with lateral acceleration control during lane changing.

**TABLE 3:** Correlation between the MoCA scores and driving measures.

	MoCA	
	*R*	P
**Lane-keeping scenario**		
SDS	−0.520	0.000
SD of throttle	−0.492	0.001
SD of acceleration	−0.531	0.000
SDLP	−0.360	0.016
**Lane-changing scenario**		
SD of lateral speed	−0.340	0.028

SD, standard deviation; SDS, standard deviation of speed; SDLP, standard deviation of lateral position; MoCA, Montreal Cognitive Assessment.

## Discussion

The purpose of this study was to investigate the driving behavior of people with insomnia under the effects of cognitive distraction in an MW state. To this end, patients diagnosed with insomnia and normal sleepers were recruited for this within-group design experiment involving two distraction tasks (a no-distraction task versus an MW task) and two driving scenarios (lane-keeping versus lane-changing). There was a significant difference in sleep quality between the CG and IG. The total MoCA scores were significantly lower in the IG than in the CG, thus, indicating the presence of significant cognitive impairment in the IG. Previous studies have found that sleep deprivation significantly reduces cognitive performance (Fuller, 2005; Zhou et al., 2011). The driving simulator experiment in this study led to several findings, as follows:

First, in the lane-keeping scenario, there was a significant interaction effect between insomnia and MW driving, leading to deficits in driving performance. In the no-distraction task in the lane-keeping scenario, there was no significant difference in longitudinal driving measures between the IG and CG. However, in the MW task, there were significant between-group differences in longitudinal driving measures; especially, the SDS, SD of acceleration, and SD of the throttle were significantly higher in the IG than in the CG. According to the task-capability interface (TCI) model, safe driving is determined by the interaction between the driver’s capability and task demand (Onate-Vega et al., 2020). Capability is referred to characteristics of the driver, such as their reaction time, motor coordination, and information processing speed, and factors that are negatively affected by human factors, such as fatigue, emotions, distractions, and drug effects (Oviedo-Trespalacios et al., 2019). Task demand is referred to the difficulty of the task, which is affected by environmental factors, such as low visibility (Chee et al., 2008). In the absence of distraction, the driving capability of both people with insomnia and normal sleepers was sufficient to meet the task demand. However, in the MW task, distraction occupied some of the driver’s cognitive resources in both the IG and CG, causing the driver to reduce the attention allocated to the driving task, thereby decreasing their capability of responding to the driving task demand. At this point, the participants in the CG were still able to meet the task demand. However, in the IG, the interaction between MW and insomnia reduced the participants’ capability to such an extent that they were no longer able to meet the driving task demand, leading to higher longitudinal control variation. The correlation analysis in this study also confirmed that the longitudinal driving performance of drivers was significantly correlated with both sleep quality and cognitive performance.

Furthermore, with respect to within-group differences, the IG showed a significantly higher SDS in the MW task than under the no-distraction task. In contrast, He et al. conducted a car-following experiment in normal sleepers and found that the SDS was lower in MW driving (He et al., 2011). The inconsistency in these results may be attributed to the greater difficulty of the car-following task, which places a higher task demand on the driver, and normal sleepers are able to compensate for deficits in speed control. Although an MW task was also performed in this study and the free driving task had a lower level of difficulty, the IG showed greater speed variation, which reflected that the people with insomnia did not show compensatory responses to deficits in driving performance. A functional magnetic resonance imaging study also found that when lapses of attention occurred during an experimental task, sleep-deprived participants showed reduced thalamic activity, which suggested a failure or impaired efficiency in the compensatory mechanisms of the cognitive control system. It is worth noting that the CG showed a higher mean speed under the more cognitively demanding MW task compared with the no-distraction task, in line with previous findings (Albert et al., 2018).

In contrast to the longitudinal driving measures, the lateral driving measures differed significantly between the IG and CG under the no-distraction task in the lane-keeping scenario, with the IG exhibiting a significantly higher SDLP than that of the CG. This is consistent with previous findings, which found that people with insomnia had more difficulty in maintaining lateral control than normal sleepers (Perrier et al., 2014). In the MW task, the between-group difference in the SDLP was significant. Notably, the SDLP in the CG was significantly higher in the MW task than in the no-distraction task, which is inconsistent with the findings of a previous report (He et al., 2011). It is possible that the presence of a vehicle in front of the driver focuses their visual attention on the road ahead, thus, improving their lane-keeping behavior and causing an inconsistency. Overall, in the lane-keeping scenario, the participants in the IG were more susceptible to the negative influence of the MW task and had more difficulty in maintaining the driving trajectory of the vehicle than those in the CG.

Second, MW had a negative effect on some lane-changing measures in both groups, although no significant between-group differences were observed for any lane-changing measures under the two distraction tasks. According to the TCI theory, our findings suggest that as the complexity of the scenarios increases (i.e., lane-changing is more demanding than lane-keeping), more cognitive resources are required, which eliminates the differences in driving control between the two groups. However, the MW-induced cognitive distraction led to varying degrees of within-group differences for both the IG and CG. Compared with the no-distraction task, the MW task resulted in a significant increase in the SD of lateral speed in the CG, as well as significant increases in the SD of the steering wheel angle, SD of lateral acceleration, and SD of lateral speed in the IG. Moreover, the correlation analysis showed that lateral speed variation was negatively correlated with participants’ cognitive function; especially, participants with lower cognitive levels showed a higher SD of lateral speed. Interestingly, increasing lateral speed variation was not significantly correlated with the participants’ sleep quality. Overall, the decrease in participants’ lane-changing control performance was predominantly associated with MW.

This study used advanced simulated technology to provide insight into the driving performance of people with insomnia, but not without some limitations. For example, the current study lacked the evaluation of psychological variables, which would reveal the internal mechanism that influences the driving behavior. Presently, there is still a lack of research on the topic. Considering the limitations of the current study, several future studies should be performed to address the following aspects. First, further research should be conducted on driving while MW takes place, as well as deciphering the influencing factors and physiological manifestations of MW while driving in patients with insomnia. Second, studies have suggested that MW is more harmful than external auditory or visual induced distractions to driving safety in normal drivers (Martens and Brouwer, 2013); however, this has not been tested in people with insomnia. Thus, future studies should also compare the effects of MW and external distraction tasks with a comparable mental load on the driving performance of people with insomnia.

## Conclusion

This study used driving simulator experiments to assess the MW distracted driving among people with insomnia and explore associated factors in various driving scenarios. The results showed that, under the lane-keeping scenario, there was no significant difference in longitudinal driving performance between the CG and IG during the no-distraction task, but the interaction effect of MW and insomnia significantly increased the drivers’ longitudinal control variant; differently, the lateral driving performance of the insomnia group was significantly worse than that of the control group under both distraction conditions. Furthermore, the driving performance was found to be favorably connected with both their level of cognition and the sleep quality. Under the lane-changing scenario, although there were no between-group driving performance differences between the two groups, MW distraction resulted in within-group variations. In addition, the driving behavior was associated with cognitive function rather than sleep quality.

## Data availability statement

The raw data supporting the conclusions of this article will be made available by the authors, without undue reservation.

## Ethics statement

The studies involving human participants were reviewed and approved by Medical Ethics Committee of General Hospital of Tianjin Medical University. Written informed consent for participation was not required for this study in accordance with the national legislation and the institutional requirements.

## Author contributions

JT and DQ designed the study. HD, YL, and ZA performed the experiments. YY analyzed the data and wrote the manuscript. LX and RX revised the manuscript. All authors have read and approved the final version of this manuscript.

## References

[B1] Ahlström C. Wachtmeister J. Nyman M. Nordenström A. Kircher K. (2020). Using smartphone logging to gain insight about phone use in traffic. *Cogn. Tech. Work* 22 181–191. 10.1007/s10111-019-00547-6

[B2] Albert D. A. Ouimet M. C. Jarret J. Cloutier M. S. Paquette M. Badeau N. (2018). Linking mind wandering tendency to risky driving in young male drivers. *Accid. Anal. Prev.* 111 125–132. 10.1016/j.aap.2017.11.019 29197692

[B3] Anderson C. Horne J. A. (2006). Sleepiness enhances distraction during a monotonous task. *Sleep* 29 573–576. 10.1093/sleep/29.4.573 16676792

[B4] Berthié G. Lemercier C. Paubel P. V. Cour M. Fort A. Galéra C. (2015). The restless mind while driving: Drivers’ thoughts behind the wheel. *Accid. Anal. Prev.* 76 159–165. 10.1016/j.aap.2015.01.005 25697452

[B5] Bharadwaj N. Edara P. Sun C. (2021). Sleep disorders and risk of traffic crashes: A naturalistic driving study analysis. *Saf. Sci.* 140:105295. 10.1016/j.ssci.2021.105295

[B6] Bhattacharya A. Morgan R. Shukla R. Ramakrishanan H. K. Wang L. (1987). Non-invasive estimation of afferent inputs for postural stability under low levels of alcohol. *Ann. Biomed. Eng.* 15 533–550. 10.1007/BF02364247 3688583

[B7] Brookhuis K. A. Volkerts E. R. O’Hanlon J. F. (1990). Repeated dose effects of lormetazepam and flurazepam upon driving performance. *Eur. J. Clin. Pharmacol.* 39 83–87. 10.1007/BF02657065 1980464

[B8] Burdett B. R. Charlton S. G. Starkey N. J. (2016). Not all minds wander equally: The influence of traits, states and road environment factors on self-reported mind wandering during everyday driving. *Accid. Anal. Prev.* 95 1–7. 10.1016/j.aap.2016.06.012 27372440

[B9] Carciofo R. Du F. Song N. Zhang K. (2014). Mind wandering, sleep quality, affect and chronotype: An exploratory study. *PLoS One* 9:e91285. 10.1371/journal.pone.0091285 24609107 PMC3946719

[B10] Carpenter J. S. Andrykowski M. A. (1998). Psychometric evaluation of the Pittsburgh Sleep Quality Index. *J. Psychosom. Res.* 45 5–13. 10.1016/s0022-3999(97)00298-5 9720850

[B11] Chee M. W. Tan J. C. Zheng H. Parimal S. Weissman D. H. Zagorodnov V. (2008). Lapsing during sleep deprivation is associated with distributed changes in brain activation. *J. Neurosci.* 28 5519–5528. 10.1523/JNEUROSCI.0733-08.2008 18495886 PMC6670628

[B12] Ferreira S. Kokkinogenis Z. Couto A. (2019). Using real-life alert-based data to analyse drowsiness and distraction of commercial drivers. *Transp. Res. F* 60 25–36. 10.1016/j.trf.2018.10.003

[B13] Fuller R. (2005). Towards a general theory of driver behaviour. *Accid. Anal. Prev.* 37 461–472. 10.1016/j.aap.2004.11.003 15784200

[B14] Galéra C. Orriols L. M’Bailara K. Laborey M. Contrand B. Ribéreau-Gayon R. (2012). Mind wandering and driving: Responsibility case-control study. *BMJ* 345:e8105. 10.1136/bmj.e8105 23241270 PMC3521876

[B15] He J. Becic E. Lee Y. C. McCarley J. S. (2011). Mind wandering behind the wheel: Performance and oculomotor correlates. *Hum. Factors* 53 13–21. 10.1177/0018720810391530 21469530

[B16] Jeon H. J. Kim J. H. Kim B. N. Park S. J. Fava M. Mischoulon D. (2014). Sleep quality, posttraumatic stress, depression, and human errors in train drivers: A population-based nationwide study in South Korea. *Sleep* 37 1969–1975. 10.5665/sleep.4252 25325495 PMC4548517

[B17] Lee H. C. Cameron D. Lee A. H. (2003). Assessing the driving performance of older adult drivers: On-road versus simulated driving. *Accid. Anal. Prev.* 35 797–803. 10.1016/S0001-4575(02)00083-0 12850081

[B18] Léger D. Massuel M. A. Metlaine A. Sisyphe Study Group. (2006). Professional correlates of insomnia. *Sleep* 29 171–178. 16494084

[B19] Lemercier C. Pêcher C. Berthié G. Valéry B. Vidal V. Paubel P. (2014). Inattention behind the wheel: How factual internal thoughts impact attentional control while driving. *Saf. Sci.* 62 279–285.

[B20] Maquet P. (2000). Functional neuroimaging of normal human sleep by positron emission tomography. *J. Sleep Res.* 9 207–231. 10.1046/j.1365-2869.2000.00214.x 11012860

[B21] Martens M. H. Brouwer R. F. T. (2013). Measuring being lost in thought: An exploratory driving simulator study. *Transp. Res. F* 20 17–28. 10.1016/j.trf.2013.04.002

[B22] Miller C. B. Robertson D. J. Johnson K. A. Lovato N. Bartlett D. J. Grunstein R. R. (2021). Tired and lack focus? Insomnia increases distractibility. *J. Health Psychol.* 26 795–804. 10.1177/1359105319842927 31007074

[B23] Miyachi T. Nomura K. Minamizono S. Sakai K. Iwata T. Sugano Y. (2021). Factors associated with insomnia among truck drivers in Japan. *Nat. Sci. Sleep* 13 613–623. 10.2147/NSS.S307904 34040470 PMC8140935

[B24] Nasreddine Z. S. Phillips N. A. Bédirian V. Charbonneau S. Whitehead V. Collin I. (2005). The Montreal cognitive assessment, MoCA: A brief screening tool for mild cognitive impairment. *J. Am. Geriatr. Soc.* 53 695–699. 10.1111/j.1532-5415.2005.53221.x 15817019

[B25] Nijboer M. Borst J. P. van Rijn H. Taatgen N. A. (2016). Driving and multitasking: The good, the bad, and the dangerous. *Front. Psychol.* 7:1718. 10.3389/fpsyg.2016.01718 27877147 PMC5100650

[B26] Onate-Vega D. Oviedo-Trespalacios O. King M. J. (2020). How drivers adapt their behaviour to changes in task complexity: The role of secondary task demands and road environment factors. *Transp. Res. F* 71 145–156. 10.1016/j.trf.2020.03.015

[B27] Oviedo-Trespalacios O. Truelove V. Watson B. Hinton J. A. (2019). The impact of road advertising signs on driver behaviour and implications for road safety: A critical systematic review. *Transp. Res. A* 122 85–98.

[B28] Perrier J. Bertran F. Marie S. Couque C. Bulla J. Denise P. (2014). Impaired driving performance associated with effect of time duration in patients with primary insomnia. *Sleep* 37 1565–1573. 10.5665/sleep.4012 25142564 PMC4153057

[B29] Philip P. Sagaspe P. Lagarde E. Leger D. Ohayon M. M. Bioulac B. (2010). Sleep disorders and accidental risk in a large group of regular registered highway drivers. *Sleep Med.* 11 973–979. 10.1016/j.sleep.2010.07.010 20961809

[B30] Rodrick D. Bhise V. Jothi V. (2013). Effects of driver and secondary task characteristics on lane change test performance. *Hum. Factors Ergon. Manuf. Serv. Ind.* 23 560–572. 10.1002/hfm.20342

[B31] Shekari Soleimanloo S. White M. J. Garcia-Hansen V. Smith S. S. (2017). The effects of sleep loss on young drivers’ performance: A systematic review. *PLoS One* 12:e0184002. 10.1371/journal.pone.0184002 28859144 PMC5578645

[B32] Smallwood J. Schooler J. W. (2006). The restless mind. *Psychol. Bull.* 132 946–958. 10.1037/0033-2909.132.6.946 17073528

[B33] Smallwood J. Mrazek M. D. Schooler J. W. (2011). Medicine for the wandering mind: Mind wandering in medical practice. *Med. Educ.* 45 1072–1080. 10.1111/j.1365-2923.2011.04074.x 21988623

[B34] Stawarczyk D. D’Argembeau A. (2016). Conjoint influence of mind-wandering and sleepiness on task performance. *J. Exp. Psychol. Hum. Percept. Perform.* 42 1587–1600. 10.1037/xhp0000254 27268466

[B35] Walker H. E. K. Trick L. M. (2018). Mind-wandering while driving: The impact of fatigue, task length, and sustained attention abilities. *Transp. Res. F* 59 81–97. 10.1016/j.trf.2018.08.009

[B36] Zhou X. Ferguson S. A. Matthews R. W. Sargent C. Darwent D. Kennaway D. J. (2011). Dynamics of neurobehavioral performance variability under forced desynchrony: Evidence of state instability. *Sleep* 34 57–63. 10.1093/sleep/34.1.57 21203373 PMC3001796

